# Effect of Different Surface Treatments on Microtensile Bond Strength of Composite Resin to Normal and Fluorotic Enamel After Microabrasion

**Published:** 2016-11

**Authors:** Mahshid Mohammadi Bassir, Mohammad Bagher Rezvani, Elham Tabatabai Ghomsheh, Zahra Malek Hosseini

**Affiliations:** 1 Assistant Professor, Department of Restorative Dentistry, Faculty of Dentistry, Shahed University, Tehran, Iran; 2 Assistant Professor, Department of Restorative Dentistry, School of Dentistry, Tehran University of Medical Sciences, Tehran, Iran; 3 Assistant Professor, Department of Restorative Dentistry, Faculty of Dentistry, Kermanshah University of Medical Sciences, Kermanshah, Iran

**Keywords:** Fluorosis, Dental, Enamel Microabrasion, Dental Bonding, Composite Resins

## Abstract

**Objectives:**

This study aimed to determine the effect of surface treatments such as tooth reduction and extending the etching time on microtensile bond strength (μTBS) of composite resin to normal and fluorotic enamel after microabrasion.

**Materials and Methods:**

Fifty non-carious anterior teeth were classified into two groups of normal and fluorotic (n=25) using Thylstrup and Fejerskov index (TFI=4–6). Teeth in each group were treated with five modalities as follows and restored with OptiBond FL and Z350 composite resin: 1-Etching (30 seconds), bonding, filling (B); 2-Tooth reduction (0.3mm), etching, bonding, filling (R-B); 3-Microabrasion (120 seconds), etching, bonding, filling (MB); 4- Microabrasion, tooth reduction, etching, bonding, filling (M-R-B); and 5- Microabrasion, etching (60 seconds), bonding, filling (M-2E-B). Ten experimental groups (n=5) were designed; 150 rectangular samples (10 in each group) with a cross-sectional area of 1×1mm^2^ were prepared for μTBS test. Failure mode was determined under a stereomicroscope and one specimen was selected from each group for scanning electron microscopy (SEM) analysis. Data were analyzed using two-way ANOVA and Tukey’s test.

**Results:**

The μTBS to normal enamel was higher than to fluorotic enamel in all groups except for group (R-B). The Maximum and minimum μTBS were noted in the group (normal, reduction, bonding) and (fluorosed, microabrasion, bonding), respectively. Tooth reduction increased μTBS more effectively than extended etching time after microabrasion.

**Conclusions:**

Fluorosis may reduce μTBS of composite resin to enamel. Microabrasion reduced the bond strength. Tooth reduction and extended etching time increased μTBS of composite resin to both normal and fluorotic enamel.

## INTRODUCTION

Fluorosis is a developmental anomaly of the enamel caused by continuous excessive fluoride consumption during tooth formation. The severity of fluorosis depends on the amount and duration of fluoride intake, individual response and bone growth during tooth development [1]. Fluorosed enamel is characterized by an outer layer of hypermineralized enamel and subsurface porosities that can exhibit white to yellow opaque spots and streaks [2].

Successful treatment of such discolorations varies from conservative to more aggressive approaches such as chemical treatment like bleaching, enamel microabrasion and composite resin or porcelain laminate veneers and crowns [3]. Microabrasion is a conservative treatment that involves the application of a paste (hydrochloric acid) and an abrasive medium (pumice) to the affected tooth surface. This treatment results in up to 0.2mm of uniform tooth surface reduction.

Microabrasion is also used for removal of enamel decalcifications that occur when plaque stays on the enamel for too long exhibiting white discolorations [4]. During microabrasion treatment, surface abrasion of enamel rods may cause by-products compacted on the microabraded surface. This newly formed enamel layer is approximately 15μm thick and has a glass-like texture called enamel glaze [2]. After microabrasion, in some patients, discolorations may not disappear completely or facial contour of tooth may alter to flat or concave shape. In these situations, composite or porcelain veneers should be considered [3].

Prior to bonding, it should be kept in mind that the micro-abraded enamel surface is more resistant to etching due to the presence of a newly formed surface layer after microabrasion. Fluorosed enamel also consists of a hypomineralized layer covered by a hypermineralized outer enamel surface that can affect bond strength and adhesion of resin to affected surface [5].

Surface treatments of micro-abraded enamel (normal and fluorotic) prior to bonding may improve the bond strength and durability of bond of composite restorations in the oral cavity. Al-Sugair and Akpata [6] reported that etching time should be extended for fluorosed enamel to achieve the typical etching pattern. Other researchers reported that surface preparation of fluorosed teeth with a diamond bur is required to remove hypermineralized surface layer prior to bonding [7,8].

Considering all the above, the present study aimed to evaluate the effect of different surface treatments on microtensile bond strength (μTBS) of composite resin to normal and fluorotic enamel after microabrasion and examine the bonding interface by light microscopy/scanning electron microscopy (SEM) for fracture analysis. The null hypotheses were that microabrasion would not reduce the bond strength and use of surface treatment prior to bonding would not improve the bond strength.

## MATERIALS AND METHODS

### Diagnosis of dental fluorosis:

The diagnosis of fluorosis was made based on 32 clinical photos of fluorosed teeth classified according to the Thylstrup and Fejerskov index (TFI) [7]. To ensure inter- and intra-examiner agreement for diagnosis of fluorosis, a reproducibility test was carried out [8]. Intra-examiner reproducibility testing yielded a Cohen’s kappa coefficient of 0.85. The calibration session was repeated several times until the inter-examiner reproducibility testing yielded a Cohen’s kappa coefficient of 0.74. Fluorosed teeth were diagnosed and categorized into six groups according to TFI (TFI 0–6).

### Collection and grouping of experimental teeth:

Fifty human anterior teeth with no caries, cracks or defects were used in this study. The 25 fluorosed teeth (TFI=4–6) were obtained from dental clinics in Qazvin, a city endemic for fluorosis. The 25 normal teeth (TFI=0) were collected from clinics in Tehran. All teeth were hand-scaled to remove tissue remnants and debris, polished with a rubber cup and a fluoride-free pumice paste and stored for up to six months in distilled water, containing 0.1 % thymol as a disinfectant in a refrigerator [9].

### Microabrasion procedure:

Microabrasion treatment was carried out using rubber cups (Opal cups, Ultradent Products, Inc., South Jordan, UT, USA) and microabrasion slurries (Opalustre, Ultradent Products, Inc., South Jordan, UT, USA) at 300 rpm in 10-second intervals for 120 seconds, using a slow contra-angle handpiece (NSK, IL, USA) and a standardized force of 100 grams equivalent to 2 bars. After surface treatment, the teeth were thoroughly rinsed to remove any remnants of slurry.

### Experimental groups:

All teeth (normal and fluorotic) were divided into 10 groups (n=5, Table 1).

**Table 1:** Experimental groups

Group 1-N	(N-B)	Normal enamel-bonding
Group 2-N	(N-R-B)	Normal enamel-reduction-bonding
Group 3-N	(N-M-B)	Normal enamel-microabrasion-bonding
Group 4-N	(N-M-R-B)	Normal enamel-microabrasion-reduction-bonding
Group 5-N	(N-M-2ET-B)	Normal enamel-microabrasion-doubled etching time-bonding
Group 6-F	(F-B)	Fluorotic enamel-bonding
Group 7-F	(F-R-B)	Fluorotic enamel-reduction-bonding
Group 8-F	(F-M-B)	Fluorotic enamel-microabrasion-bonding
Group 9-F	(F-M-R-B)	Fluorotic enamel-microabrasion-reduction-bonding
Group 10-F	(F-M-2ET-B)	Fluorotic enamel-microabrasion-doubled etching time-bonding

- In groups 1 and 6 [bonding (B)] adhesive (OptiBond FL, Kuraray, Tokyo, Japan) was applied according to the manufacturer's instructions with 15 seconds of etching time and then samples were rinsed with water for 15 seconds and slowly dried for five seconds.
  Primer was applied on the surface and after that adhesive was applied and gently dried. Adhesive was cured for 30 seconds (Table 2).
-In groups 2 and 7 [reduction, bonding (R-B)], first 0.3mm deep standard enamel reduction was performed using a diamond bur with depth orientation groove (No: 834; Komet, Lemgo, Germany) in the labial surface of the teeth. The reduction was completed using 012 round-end taper diamond bur (Brasseler USA, Savannah, GA, USA.). After that, the adhesive was applied according to the manufacturer's instructions.
-In groups 3 and 8 [microabrasion, bonding (MB)], adhesive was applied after microabrasion treatment.
-In groups 4 and 9 [microabrasion, reduction, bonding (M-R-B)], after microabrasion, 0.3mm of tooth surface was removed by diamond bur and then the adhesive was applied according to the manufacturer’s instructions.
-In groups 5 and 10 [microabrasion, doubled etching time, bonding (M-2E-B)], after microabrasion, etchant was applied for up to 60 seconds followed by the application of adhesive similar to what was done in the other groups. All the teeth were shaped in a flat rectangular form for bond to composite resin for μTBS testing.

**Table 2:** Materials used in this study

** Material **	** Type **	** Main Composition **	** Manufacturer **
Filtek Z350	Nano composite resin	Combination of aggregated zirconia/silica cluster filler, bis-GMA, UDMA, TEGDMA, bis-EMA	3M ESPE, St. Paul, MN, USA
OptiBond FL	Adhesive resin	Etchant: 37% phosphoric acid Primer: HEMA, GPDM, ethanol, BHT, water, PAMA, CQ Adhesive: Bis-GMA, HEMA, GDMA, CQ	Kerr, Orange, CA, USA
Opalustre	Microabrasion paste	Acid: 6.6% hydrochloric acid Abrasive: Pumice (silicon carbide)	Ultradent, Jordan, South UT, USA

Bis-GMA: Bis-Phenol A glycidyl methacrylate; UDMA: Urethane dimethacrylate; TEGDMA: Triethylene glycol dimethacrylate; Bis-EMA: Bisphenol A-polyethylene glycol diether dimethacrylate; HEMA: Hydroxy ethyl methacrylate; CQ: Comphorquinone

Composite resin (Z350; 3M ESPE, St. Paul, MN, USA) was applied to the enamel surface in six increments with a thickness of 6mm, and each layer was light cured for 20 seconds. Light-curing was performed using Optilux 500 light curing unit (Demetron; Kerr, Danbury, CT, USA) with a light intensity of 560 mW/cm ^2^ . The intensity of the curing light was measured by a radiometer and the recorded intensity was 560 mW/cm ^2^ .

### Microtensile bond strength testing:

After the bonding procedure, all samples were stored in tap water at 37°C for 24 hours. The teeth were sectioned with a water-cooled slow-speed diamond saw (Isomet 1000; Buehler Ltd., Lake Bluff, IL, USA) perpendicular to the bonding surface in both x and y directions to obtain three rectangular resin-enamel sticks measuring 1×1mm ^2^ . The dimensions of the sticks were measured by means of a digital caliper (CD-15 S; Mitutoyo, Tokyo, Japan). The non-trimmed samples were fixed to modified Geraldeli μTBS testing jig [10] with cyanoacrylate glue (Model Repair II blue, Dentsply-Sankin, Tokyo, Japan) and loaded in the LRX testing machine (Lloyd, Hampshire, UK) with a 100N load cell and a crosshead speed of 1 mm/minute.

Samples that failed before actual testing (pre-testing failure) were excluded from the study. The μTBS was calculated and reported in MPa by dividing the applied force (N) at the time of fracture by the bonding area (mm ^2^). Statistical analysis was performed using SPSS version 18 software (SPSS Inc., IL, USA). Two-way ANOVA and post hoc Tukey’s HSD multiple comparisons test were used to determine statistical differences in μTBS between the groups. P<0.05 was considered statistically significant.

### Failure analysis:

The mode of failure was evaluated under light microscopy at ×50 magnification using a stereomicroscope (Wild MSA, Heerbrugg, Switzerland). One sample from each group was selected and observed with Fe-SEM (Philips, XL30, Eindhoven, Netherland), using electron microscopy specimen processing techniques including fixation, dehydration, chemical drying and gold-sputter coating. The failure mode was classified as interfacial failure between the tooth structure and adhesive resin (type 1), cohesive failure of composite resin (type 2), cohesive failure of adhesive resin (type 3), cohesive failure of enamel (type 4) and mixed adhesive-cohesive failure (type 5).

## RESULTS

### Microtensile bond strength:

Table 3 shows the mean μTBS, standard deviation (SD), and number of samples in the groups. The maximum μTBS was found in the (normal, reduction, bonding) group and the minimum μTBS was found in the (fluorosed, microabrasion, bonding) group. The μTBS of fluorosed teeth was significantly lower than that of normal teeth (fluorotic teeth: 37.4MPa, normal teeth: 40.6MPa, P=0.00). Surface preparation with diamond bur significantly increased the μTBS in both types of enamel (fluorotic teeth: 48.7MPa, normal teeth: 49.7MPa, P=0.00). Microabrasion treatment significantly decreased the μTBS in both types of enamel (fluorotic teeth: 19.2MPa, normal teeth: 24.0 MPa, P=0.00). Surface treatments increased the μTBS to micro-abraded enamel in both enamel types (fluorotic teeth: 45.45MPa, normal teeth: 47.3MPa, P=0.00). The reduction of microabraded enamel increased μTBS significantly more than extending the etching time (reduction: 47.35MPa, extended etching time: 45.4 MPa, P=0.00). For normal enamel, no significant difference was detected in μTBS after reduction with or without microabrasion (P>0.05). But in fluorosed enamel, μTBS after reduction was lower in micro-abraded enamel compared to the enamel surface that had not undergone microabrasion (P=0.00).

**Table 3:** The mean microtensile bond strength (megapascals) in all groups

** Groups **	** SD **	** Mean **	** Min **	** Max **
N-B-1	1.2	34.7	31.77	36.76
N-R-B-2	1.9	49.7a	46.88	53.37
N-M-B-3	0.6	24.0	22.86	24.95
N-M-R-B-4	1.8	48.3a	43.84	51.30
N-M-2E-B-5	1.4	46.3	44.40	49.31
F-B-6	1.2	28.2	26.63	31.08
F-R-B-7	1.3	48.7	46.44	51.47
F-M-B-8	0.8	19.2	17.55	21.12
F-M-R-B-9	1.6	46.4	43.54	50.00
F-M-2E-B-10	1.9	44.5	41.00	48.42

* Lower case letters: the means were not statistically different.

N: Normal; B: Bonding; R: Reduction; M: Microabrasion; E: Etching Time; F: Fluorotic

### Failure analysis:

**Fig. 1:**
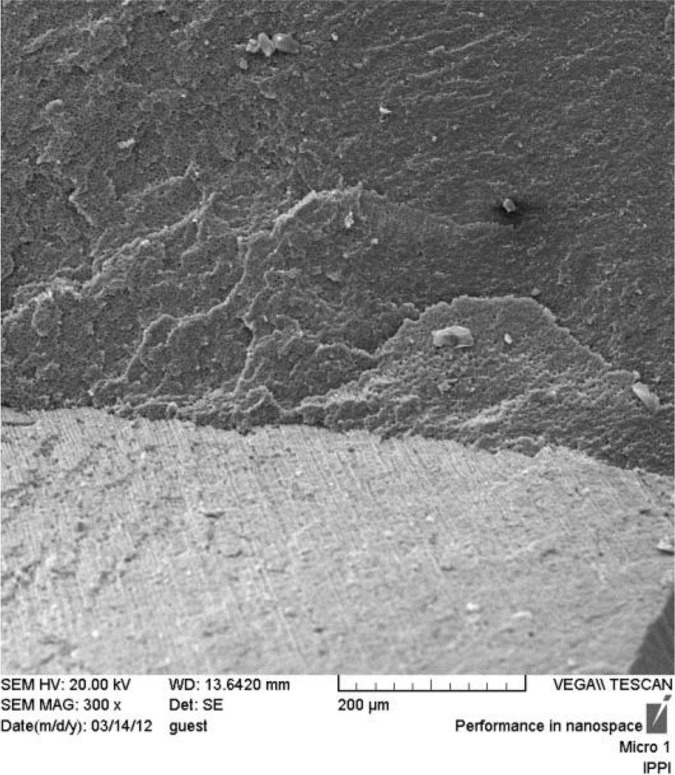
SEM micrograph of adhesive failure

**Fig. 2:**
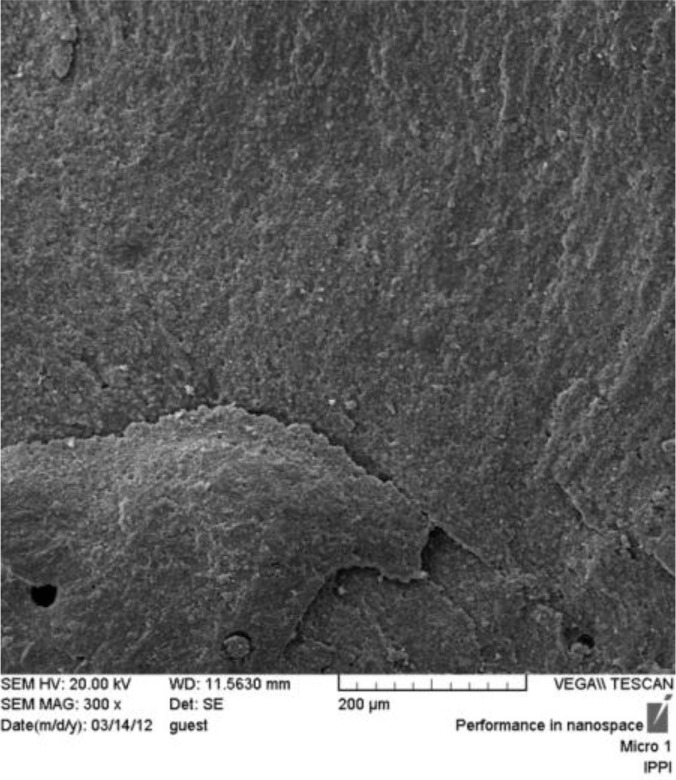
SEM micrograph of cohesive failure in composite

**Fig. 3:**
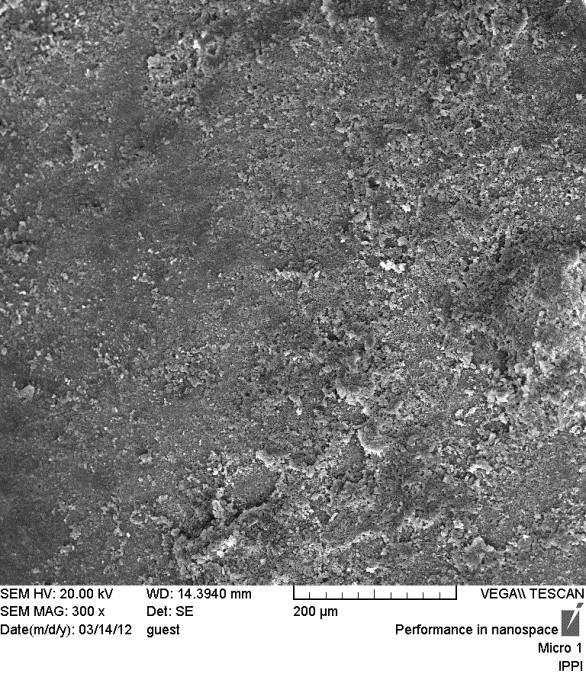
SEM micrograph of cohesive failure in enamel

The failure modes of all samples are reported in Figures 1 to 3 and Table 4. Most failures were recorded as mixed (n=76), irrespective of enamel type and experimental condition.

**Table 4:** Failure modes

** Groups **	** Type 1 **	** Type 2 **	** Type 3 **	** Type 4 **	** Type 5 **	** Total **
** (N-B) **	3	0	3	2	7	15
** (N-R-B) **	0	1	2	1	11	15
** (N-M-B) **	2	2	3	2	6	15
** (N-M-R-B) **	0	1	2	2	10	15
** (N-M-2ET-B) **	0	3	3	2	7	15
** (F-B) **	2	5	2	1	5	15
** (F-R-B) **	1	3	2	0	9	15
** (F-M-B) **	3	4	2	0	6	15
** (F-M-R-B) **	0	2	3	2	8	15
** (F-M-2ET-B) **	0	3	2	3	7	15
** Total **	11	2	24	15	76	150

In both types of enamel, samples had an interfacial failure pattern when the adhesive was bonded to unground enamel or micro-abraded enamel without reduction. Fluorosed teeth tended to fail more cohesively in the enamel (n=17) than the teeth without fluorosis (n=7).

## DISCUSSION

The first null hypothesis was refuted since microabrasion treatment reduced the bond strength of composite resin to tooth surface. The second null hypothesis was also rejected as the use of surface treatment prior to bonding improved the bond strength.

Structural differences between normal and fluorotic teeth can influence the bond strength of composite resins to enamel. Fluorosed and normal teeth in this study were classified according to the TFI, which is based on histopathological changes in relation to clinical manifestations of fluorosed teeth [7]. The teeth classified as TFI =4–6 appear to be chalky and show distinct pit hole defects. Also, sometimes the deep subsurface lesions necessitate the use of a more complex treatment such as composite or porcelain laminate veneering to achieve optimal esthetic results [11].

Al-Sugair and Akpata [6] showed that the aforementioned outer hypermineralized layer is highly resistant to acid-etching. Ermis et al, [8] also indicated that bonding effectiveness to unground fluorosed enamel was lower than to normal teeth because fluorapatite in the outer enamel surface is more crystalline and more stable; thus, it resists dissolution in acid-etchant. For these reasons, it seems that structural changes in fluorotic enamel with hypermineralized outer layer can interfere with bond strength of composite resin to fluorosed enamel.

Enamel microabrasion, which is defined as a combination of abrasion and erosion, apparently reduces the superficial enamel thickness yielding a dense, prismless and resistant layer [5]. This layer is comprised of a compact mineralized tissue within an organic matrix, replacing the prism-rich enamel with a densely compacted prism-free layer [12]. It seems that microabrasion can reduce bond strength of composite resin to enamel by altering enamel surface structure.

Croll et al, [12] found that by increased severity of fluorosis, the lesions extend towards the inner enamel to inner half of the enamel thickness. It seems that the subsurface hypomineralized porous layer results in greater compaction of microabrasion by-products into the fluorotic compared to normal enamel surface; thus, the bond strength of composite resin decreases more significantly to fluorosed enamel after microabrasion [13].

Opinya and Pameijer [14] observed that grinding the outer hypermineralized surface layer can result in higher bond strength to composite resin. Ermis et al, [8] also showed that 0.3mm reduction of enamel surface can increase the tensile bond strength to fluorosed teeth. Some authors have suggested that fluorapatite in the outer layer of fluorosed enamel is more resistant to acid dissolution than the hydroxyapatite in normal teeth and thus the etching time of fluorosed enamel should be doubled [6,14].

In this study, grinding and extending the etching time increased bond strength of composite resin to micro-abraded enamel but the normal enamel still showed a higher level of bond strength in comparison to fluorotic enamel. It seems that the higher concentration of organic compounds and deeper extension of micro-pore defects into the fluorotic enamel still affected the bond strength although the micro-abraded layer had been ground [13].

Failure mode analysis showed that fluorotic enamel in all groups had a higher prevalence of cohesive failure of enamel, which is attributed to the structure of fluorotic enamel. Mixed failure had the highest prevalence among the modes of failure in all groups indicating that acceptable level of bond strength was achieved after surface preparation.

In this study, reduction of enamel surface increased the bond strength of composite resin to fluorosed enamel as high as the level of bond to normal enamel. This is in agreement with the findings of Ermis et al, [8] who showed that preparation of enamel before the bonding procedure improved the resin-enamel bond strength in fluorosed teeth.

In normal teeth, it seems that reduction of enamel surface after microabrasion improved the bond strength to the level of not-abraded enamel. In fluorotic teeth, grinding the micro-abraded enamel improved the bond strength but not to the level of not-abraded enamel. It seems that in fluorotic teeth, the subsurface porosities are so deep that they cause the compaction of microabrasion by-products deep into the enamel surface and reduce the bond strength of composite resin to micro-abraded fluorotic enamel in comparison to non-abraded enamel surface. In normal teeth, no significant difference was detected between the two methods of surface treatment (grinding and extended etching time) in improving the tensile bond strength. But, in fluorotic teeth, grinding improved the bond strength of composite resin significantly greater than extending the etching time. The initial effect of phosphoric acid on enamel etching is to remove 10μm of superficial enamel. The differential dissolution of enamel rods and inter-rods forms 20μm-deep micromechanical retentions [15]. Since microabrasion forms a 15-20μm thick layer on enamel surface [5], it seems that extending the etching time cannot thoroughly eliminate this layer resulting in lower bond strength in comparison to grinding of fluorotic teeth.

Considering the limitations of this study, it seems that future research is needed to assess the effect of different surface treatments on μTBS of composite resins to fluorosed teeth with different severities. Complementary experiments such as microhardness and histochemical analysis could also be helpful for a more precise assessment.

## CONCLUSION

The bonding efficacy to fluorosed teeth was lower than that to normal teeth. Microabrasion treatment reduced bond strength to both types of enamel. Surface preparation improved bond strength to both types of enamel. Grinding improved the bond strength more than extending the etching time.
